# *Interleukin 4* gene polymorphism (−589C/T) and the risk of asthma: a meta-analysis and met-regression based on 55 studies

**DOI:** 10.1186/s12865-020-00384-7

**Published:** 2020-10-21

**Authors:** Ahmad Kousha, Armita Mahdavi Gorabi, Mehdi Forouzesh, Mojgan Hosseini, Markov Alexander, Danyal Imani, Bahman Razi, Mohammad Javad Mousavi, Saeed Aslani, Haleh Mikaeili

**Affiliations:** 1 Department of Health Education and Health Promotion, Faculty of Health, Tabriz University of Medical Sciences, Tabriz, Iran; 2 Research Center for Advanced Technologies in Cardiovascular Medicine, Tehran Heart Center, Tehran University of Medical Sciences, Tehran, Iran; 3 Legal medicine Research Center, Legal Medicine organization, Tehran, Iran; 4 Department of Science, Islamshahr Branch, Islamic Azad University, Sayad Shirazi St. Islamshahr, Tehran, Iran; 5 Tyumen State Medical University, Tyumen, Russian Federation; 6 Department of Immunology, School of Public Health, Tehran University of Medical Sciences, Tehran, Iran; 7 Department of Hematology and Blood Banking, School of Medicine, Tarbiat Modares University, Tehran, Iran; 8 Department of Hematology, Faculty of Allied Medicine, Bushehr University of Medical Sciences, Bushehr, Iran; 9 Department of Immunology, School of Medicine, Tehran University of Medical Sciences, Tehran, Iran; 10 Tuberculosis and Lung Diseases Research Center, Tabriz University of Medical sciences, Tabriz, Iran

**Keywords:** Asthma, Interleukin 4, Polymorphism, Meta-analysis, Genetic susceptibility

## Abstract

**Background:**

Numerous investigations have previously evaluated the association of *interleukin (IL) 4* gene polymorphisms and the risk of asthma, conferring inconsistent results. To resolve the incongruent outcomes yielded from different single studies, we conducted the most up-to-date meta-analysis of *IL4* gene −589C/T (rs2243250) polymorphism and susceptibility to asthma.

**Methods:**

A systematic literature search was performed in ISI web of science, Scopus, Medline/PubMed databases prior to September 2020, and the pooled odds ratio (OR) and their corresponding 95% CI were calculated to determine the association strength.

**Results:**

Literature search led to retrieving of 49 publications (55 case-control studies) containing 9572 cases and 9881 controls. It was revealed that *IL4* gene −589C/T polymorphism increased the risk of asthma across all genetic models, including dominant model (OR = 1.22), recessive model (OR = 1.17), allelic model (OR = 1.21), and TT vs. CC model (OR = 1.34), but not the CT vs. TT model. The subgroup analysis by age indicated that *IL4* gene -589C/T polymorphism was significantly associated with asthma risk in both pediatrics and adults. Additionally, the subgroup analysis by ethnicity revealed significant association in Asian, American, and Europeans. Finally, subgroup analysis by East Asian and non-East Asian populations indicated significant associations.

**Conclusions:**

The current meta-analysis revealed that *IL4* gene -589C/T polymorphism was a susceptibility risk in both pediatrics and adults in the whole and different ethnic groups.

## Background

Asthma is one of the most common atopic disorders of the respiratory tract, which results in wheezing, coughing, breathlessness, and bronchial obstruction [1]. The prevalence and incidence of asthma raised regularly and it estimated more than 300 million persons of the world are affected by this disease [2]. The main causes of asthma are not completely clear. However, is has been postulated that asthma is mediated by interactions between specific external stimulating factors, including pollutants, viral and bacterial infections, allergens, tobacco smokes, etc., and genetics of the patients. Additionally, increasing number of studies recommend that genetic factors play a critical role in the etiology of asthma by their interactions with the environmental elements [3, 4]. The heritability of asthma is estimated to be 35 to 95% [5]. Numerous studies have examined the correlation between genetic variations of pro and anti-inflammatory genes and susceptibility to asthma [6, 7]. In recent decades, single nucleotide polymorphisms (SNP) have become one of the frequently studied models of DNA variation in analyses of the association between genetics and susceptibility to disease [8, 9].

The role of immunological factors especially cytokines in modulating and controlling the inflammatory response of the respiratory tracts is essential in the evolution, progression, and exacerbations of asthma [10]. Interleukin (IL)-4 is a key ingredient of the immune system required in the regulation of response to an allergen through controlling the isotype switching of antibody in B lymphocytes to IgG and IgE class [11]. Elevated serum levels of IgE are suggestive of allergic reactions and resemble a high level of IL-4 mRNA assembly [12]. Moreover, it acts as a growth factor to facilitate the differentiation of T helper (Th) 2 cells and mast cells. These characteristics of IL-4 accentuate on the crucial roles of cytokines in the pathogenesis asthma [13, 14]. Additionally, *IL4* gene polymorphisms, like promoter region (C + 33 T) SNP [15], and 3017 G/T SNP in intron 2 [16], have been associated with IgE levels, which might be involved in the pathogenesis of asthma.

The *IL4* gene is located on chromosome 5q31 [17]. The -589C/T (rs2243250) polymorphism has been recognized on upstream of the transcription initiation site [18]. It has been demonstrated that the binding of a transcription factor is enhanced by the appearance of the polymorphic T allele that may result in an overexpression of the *IL4* gene and, thus, raising the power of any immunological response that dependents on IL-4 [19]. To date, many studies have examined the association between IL4 gene -589 C/T polymorphisms and the risk of asthma, but their outcomes have not been consistent. Therefore, we performed this meta-analysis to analyze the relationship between the -589C/T polymorphisms and susceptibility to asthma.

## Methods

This study conducted in accordance with the Preferred Reporting Items for Systematic reviews and Meta-Analyses (PRISMA) statement including; literature review, study selection, inclusion and exclusion criteria, data extraction and quality assessment, and statistical analysis [20]. No ethics committee confirmation was necessary for this meta-analysis, which does not contain any studies with human participants or animals performed by any of the authors.

### Literature review

A comprehensive search was performed in the ISI web of science, Scopus, Medline/PubMed databases to retrieve published articles prior to September 2020. The following main key words and Medical Subject Headings (Mesh) were searched: (“asthma” [Mesh] OR “asthmatic”) AND (“interleukin-4” OR “IL-4” OR “rs2243250”) AND (“single nucleotide polymorphism” OR “SNP” OR “polymorphisms” OR “mutation” OR “variation”). No restrictions were placed on language, sample size, population or publication date.

### Study selection

The retrieved publications by primary literature search were imported into Endnote X8 software. The duplicate studies were removed and title and abstract of remain studies were reviewed by two investigators. Full-text verification was performed if we could not categorize studies based on title and abstract. Any disagreements during study selection was discussed and resolved by consensus.

### Inclusion and exclusion criteria

The following inclusion criteria were used to distinguish eligible studies: i) studies with distinct case and control group evaluating the association between IL-4 C589T polymorphism and susceptibility to asthma; ii) studies with calculable or extractable data for odds ratio (OR) and 95% confidence intervals (CIs); iii) studies with sufficient data for alleles and genotypes in case and control group. The duplicates, reviews, book chapters, and meta-analysis were excluded. The application of these criteria results in 49 qualified studies for the meta-analysis.

### Data extraction and quality assessment

Two of our authors independently and according to an extraction checklist extracted the following data: the first author, journal and year of publication, country of origin, ethnicity, number of subjects in the case and the control groups for each gender, mean or range of age, genotyping method, genotype counts in the case and the control group. The quality of each study was assessed using the Newcastle-Ottawa Scale (NOS) criteria [21]. Studies with scores 0–3, 4–6 or 7–9 were low, moderate or high-quality, respectively.

### Statistical analysis

Statistical analyses were carried out using STATA (version 14.0; Stata Corporation, College Station, TX) and SPSS (version 23.0; SPSS, Inc. Chicago, IL). The strength of association between polymorphism and asthma susceptibility was estimated by odd ratios (ORs) and 95% confidence intervals (CIs) for the dominant model, recessive model, allele contrasts, and additive comparison. Heterogeneity among included studies was measured via Q statistics (*P* value< 0.1 considered statistically significant) and I^2^-test (I^2^ values of 25, 50 and 75% were described as low, moderate, and high heterogeneity, respectively). In the presence of heterogeneity random effect model (REM) was used, however fixed effect model (FEM) was applied in homogeneous condition [22, 23]. In order to assessed the predefined sources of heterogeneity among included studies, subgroup analysis and meta-regression analysis based on year of population, the continent of the study population, and genotyping method were performed. The genotypic frequency distribution in the controls was checked for consistency of the Hardy– Weinberg equilibrium (HWE). Furthermore, publication bias was computed by the Begg’s and Egger’s test and visual examination of the funnel plot (*P* value< 0.05 considered statistically significant) [24, 25]. Additionally, to calculate overall effect size in absence of each study, a sensitivity analysis was conducted.

## Results

### Search results and characteristics of the selected studies

**Fig. 1:**
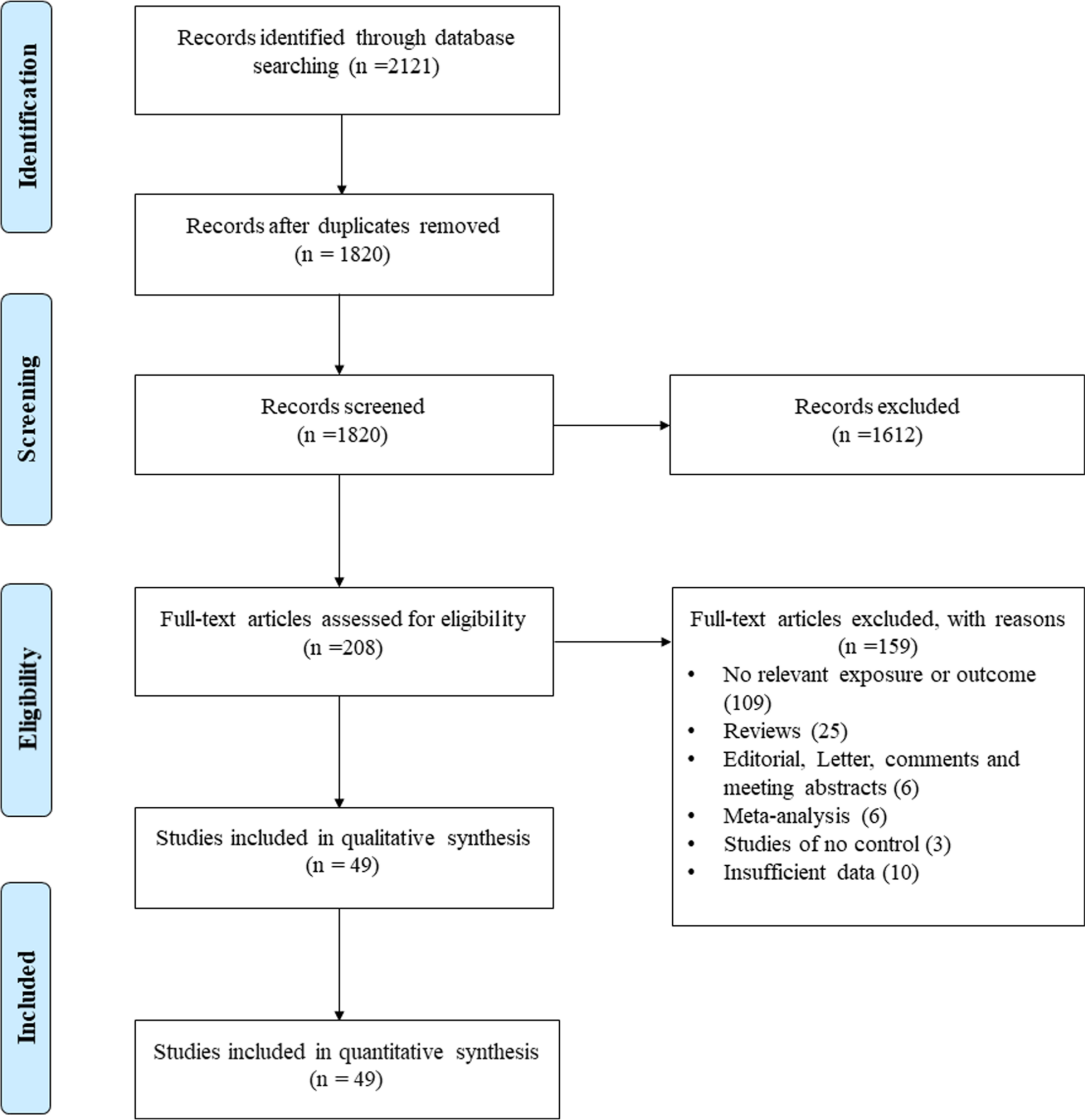
Flow diagram of study selection process

Our primary search retrieved 2121 potential articles. After removing of duplicate articles (*n* = 301), 1820 articles remain for abstract and full-text screening. Of 1820 articles, 1612 were excluded base on title and abstract and 159 articles based on full-text reading. Ultimately 49 publications with 9579 cases and 9881 controls met the inclusion criteria and their data were extracted for meta-analysis. Among these 49 publications, four of them, including Basehore et al. [16], Donfack et al. [26], Zhang et al. [27], and Baye et al. [28] examined two or three different populations with separate case and control; therefore, we assumed them as 9 case-control studies collectively (55 studies). The detailed information on study selection process is illustrated in Fig. 1, Tables 1, and 2.

**Table 1:** Characteristics of studies included in meta-analysis of overall asthma

Study author	Year	Country	Ethnicity 1 (Continent)	Ethnicity 2	Ethnicity 3	Age group	Total cases/control	Genotyping method	Quality Score
Walley et al. [29]	1996	UK	Europe	non East-Asian	Caucasian	Pediatric	124 / 59	PCR-RFLP	6
Hijazi et al. [30]	2000	Kuwait	Asia	non East-Asian	Arab	Mixed	84 / 100	PCR-RFLP	6
Sandford et al. [31]	2000	New Zealand	Europe	non East-Asian	Caucasian	Adult	233 / 143	PCR-RFLP	7
Takabayashi et al. [32]	2000	Japan	Asia	East-Asian	Caucasian	Pediatric	100 / 100	PCR-RFLP	6
Hakonarson et al. [33]	2001	Iceland	Europe	non East-Asian	Caucasian	Mixed	94 / 94	PCR	6
Cui et al. [34]	2003	China	Asia	East-Asian	Caucasian	Mixed	241 / 175	PCR-RFLP	7
Basehore et al. (i) [16]	2004	USA	America	non East-Asian	African American	Adult	233 / 245	PCR	7
Basehore et al. (ii) [16]	2004	USA	America	non East-Asian	African American	Adult	168 / 269	PCR	7
Basehore et al. (iii) [16]	2004	USA	America	non East-Asian	African American	Adult	116 / 130	PCR	6
Lee et al. [35]	2004	Korea	Asia	East-Asian	Caucasian	Pediatric	254 / 100	PCR-RFLP	6
Park et al. [36]	2004	Korea	Asia	East-Asian	Caucasian	Mixed	532 / 170	SNaPshot	8
Wang et al. [37]	2004	China	Asia	East-Asian	Caucasian	Adult	93 / 62	PCR-RFLP	6
Adjers et al. [38]	2004	Finland	Europe	non East-Asian	Caucasian	Adult	243 / 401	PCR-RFLP	7
Donfack et al. (i) [26]	2005	USA	America	non East-Asian	African American	Mixed	126/ 205	LAS	6
Donfack et al. (ii) [26]	2005	USA	America	non East-Asian	African American	Mixed	205 / 183	LAS	7
Zhang et al. (i) [27]	2005	China	Asia	East-Asian	Caucasian	Adult	152 / 157	PCR-RFLP	6
Zhang et al. (ii) [27]	2005	Malaysia	Asia	East-Asian	Caucasian	Adult	76 / 100	PCR-RFLP	6
Zhang et al. (iii) [27]	2005	India	Asia	non East-Asian	Caucasian	Adult	87 / 103	PCR-RFLP	6
Gervaziev et al. [39]	2006	Russia	Europe	non East-Asian	Caucasian	Adult	109 / 68	PCR-RFLP	6
Schubert et al. [40]	2006	Germany	Europe	non East-Asian	Caucasian	Pediatric	231 / 270	PCR-RFLP	7
Kabesch et al. [41]	2006	Germany	Europe	non East-Asian	Caucasian	Pediatric	73 / 773	PCR-RFLP	6
Battle et al. [42]	2007	USA	America	non East-Asian	African American	Mixed	255 / 175	PCR-RFLP	6
Hosseini-Farahabadi et al. [43]	2007	Iran	Asia	non East-Asian	Caucasian	Adult	30 / 50	PCR-RFLP	5
Kamali-Sarvestani et al. [44]	2007	Iran	Asia	non East-Asian	Caucasian	Adult	149 / 112	PCR-RFLP	6
Chiang et al. [45]	2007	China	Asia	East-Asian	Caucasian	Adult	167 / 111	PCR-RFLP	6
Mak et al. [46]	2007	China	Asia	East-Asian	Caucasian	Adult	289 / 292	PCR-RFLP	7
Attab et al. [47]	2008	Jordan	Asia	non East-Asian	Arab	Pediatric	40 / 40	PCR-RFLP	5
De Faria et al. [48]	2008	Brazil	America	non East-Asian	Caucasian	Pediatric	88 / 202	PCR-RFLP	6
Jiang et al. [49]	2009	China	Asia	East-Asian	Caucasian	Adult	13 / 13	PCR-RFLP	5
Amirzargar et al. [50]	2009	Iran	Asia	non East-Asian	Caucasian	Mixed	59 / 139	PCR-RFLP	6
Daley et al. [51]	2009	Australia	Oceania	non East-Asian	Caucasian	Mixed	644 / 751	Illumina Bead array system	8
Haller et al. [52]	2009	USA	America	non East-Asian	African American	Adult	72 / 70	PCR-RFLP	6
Rad et al. [53]	2010	Iran	Asia	non East-Asian	Caucasian	Adult	64 / 65	PCR-RFLP	6
Wu et al. [54]	2010	China	Asia	East-Asian	Caucasian	Pediatric	252 / 227	PCR-RFLP	7
Beghe et al. [55]	2010	UK and Italy	Europe	non East-Asian	Caucasian	Mixed	299 / 176	PCR-RFLP	7
Bijanzadeh et al. [56]	2010	India	Asia	non East-Asian	Caucasian	Mixed	100 / 50	PCR-RFLP	6
Fance et al. [57]	2010	China	Asia	East-Asian	Caucasian	Adult	62 / 30	PCR-RFLP	6
Baye et al. (i) [28]	2011	USA	America	non East-Asian	African American	Pediatric	413 / 298	Illumina GoldenGate Assay system	7
Baye et al. (ii) [28]	2011	USA	America	non East-Asian	African American	Pediatric	315 / 51	Illumina GoldenGate Assay system	6
Daneshmandi et al. [58]	2011	Iran	Asia	non East-Asian	Caucasian	Adult	81 / 124	PCR-RFLP	7
Huang et al. [59]	2011	China	Asia	East-Asian	Caucasian	Pediatric	100 / 122	PCR-RFLP	6
Hwang et al. [60]	2012	China	Asia	East-Asian	Caucasian	Pediatric	188 / 376	PCR-RFLP	7
Chiang et al. [61]	2012	China	Asia	East-Asian	Caucasian	Adult	452 / 106	PCR-RFLP	6
Micheal et al. [62]	2013	Pakistan	Asia	non East-Asian	Caucasian	Mixed	108 / 120	PCR-RFLP	6
Ricciardolo et al. [63]	2013	Italy	Europe	non East-Asian	Caucasian	Mixed	57 / 124	PCR-SSP	6
Smolnikova et al. [64]	2013	Russia	Europe	non East-Asian	Caucasian	Mixed	64 / 50	PCR-RFLP	6
Li et al. [65]	2014	China	Asia	East-Asian	Caucasian	Pediatric	491 / 503	PCR-LDR	7
Wang et al. [66]	2015	China	Asia	East-Asian	Caucasian	Mixed	392 / 849	Mass array	7
Dahmani et al. [67]	2016	Algeria	Africa	non East-Asian	Arab	Adult	44 / 19	PCR-RFLP	6
Li et al. [68]	2016	China	Asia	East-Asian	Caucasian	Pediatric	317 /351	PCR and Sequencing	7
Narozna et al. [69]	2016	Poland	Europe	non East-Asian	Caucasian	Mixed	177 / 189	Taq Man	7
Zhang et al. [68]	2016	China	Asia	East-Asian	Caucasian	Pediatric	38 / 35	PCR and Sequencing	6
Hussein et al. [70]	2017	Iraq	Asia	non East-Asian	Arab	Mixed	48 / 25	ARMS-PCR	6
Abood et al. [71]	2018	Iraq	Asia	non East-Asian	Arab	Mixed	100 / 100	AS-PCR	6
Zhang et al. [72]	2019	China	Asia	East-Asian	Caucasian	Pediatric	37 / 29	PCR and Sequencing	5

**Table 2:** Distribution of genotype and allele among asthma patients and controls

Study author	Asthma cases					Healthy control					P-HWE	MAF
	CC	CT	TT	C	T	CC	CT	TT	C	T		
Walley et al. [29]	56	55	13	167	81	31	23	5	85	33	0/8	0/72
Hijazi et al. [30]	5	25	54	35	133	9	31	60	49	151	0/1	0/245
Sandford et al. [31]	146	78	9	370	96	100	41	2	241	45	0/33	0/842
Takabayashi et al. [32]	6	43	51	55	145	10	39	51	59	141	0/53	0/295
Hakonarson et al. [33]	73	20	1	166	22	67	25	2	159	29	0/85	0/845
Cui et al. [34]	11	89	141	111	371	9	52	114	70	280	0/34	0/2
Basehore et al. (i) [16]	153	72	8	378	88	181	59	5	421	69	0/94	0/859
Basehore et al. (ii) [16]	22	77	69	121	215	29	119	121	177	361	0/97	0/329
Basehore et al. (iii) [16]	43	55	18	141	91	55	59	16	169	91	0/97	0/65
Lee et al. [35]	9	77	168	95	413	3	29	68	35	165	0/96	0/175
Park et al. [36]	19	164	349	202	862	7	54	109	68	272	0/92	0/2
Wang et al. [37]	29	42	22	100	86	21	26	15	68	56	0/22	0/548
Adjers et al. [38]	106	103	34	315	171	189	164	48	542	260	0/18	0/675
Donfack et al. (i) [26]	85	34	7	204	48	144	55	6	343	67	0/78	0/836
Donfack et al. (ii) [26]	25	82	98	132	278	24	82	77	130	236	0/76	0/355
Zhang et al. (i) [27]	4	47	101	55	249	3	45	109	51	263	0/5	0/162
Zhang et al. (ii) [27]	11	35	30	57	95	16	43	41	75	125	0/4	0/375
Zhang et al. (iii) [27]	50	31	6	131	43	66	30	7	162	44	0/17	0/786
Gervaziev et al. [39]	16	75	18	107	111	18	43	7	79	57	0/01	0/58
Schubert et al. [40]	143	78	10	364	98	189	74	7	452	88	0/93	0/837
Kabesch et al. [41]	42	29	2	113	33	564	188	21	1316	230	0/26	0/851
Battle et al. [42]	28	113	114	169	341	19	77	79	115	235	0/97	0/328
Hosseini-Farahabadi et al. [43]	17	8	5	42	18	38	12	0	88	12	0/33	0/88
Kamali-Sarvestani et al. [44]	139	6	4	284	14	93	18	1	204	20	0/9	0/91
Chiang et al. [45]	1	19	147	21	313	7	34	70	48	174	0/31	0/216
Mak et al. [46]	15	95	179	125	453	19	87	186	125	459	0/05	0/214
Attab et al. [47]	31	9	0	71	9	33	7	0	73	7	0/54	0/912
De Faria et al. [48]	38	41	9	117	59	67	108	27	242	162	0/1	0/599
Jiang et al. [49]	0	8	5	8	18	1	9	3	11	15	0/13	0/423
Amirzargar et al. [50]	0	59	0	59	59	10	129	0	149	129	< 0.001	0/535
Daley et al. [51]	476	155	13	1107	181	549	186	16	1284	218	0/95	0/854
Haller et al. [52]	6	30	36	42	102	7	31	32	45	95	0/89	0/321
Rad et al. [53]	46	18	0	110	18	42	23	0	107	23	0/08	0/823
Wu et al. [54]	6	83	163	95	409	11	84	132	106	348	0/61	0/233
Beghe et al. [55]	232	63	4	527	71	136	37	3	309	43	0/79	0/877
Bijanzadeh et al. [56]	92	4	4	188	12	48	1	1	97	3	< 0.001	0/97
Fance et al. [57]	38	13	11	89	35	27	1	2	55	5	< 0.001	0/916
Baye et al. (i) [28]	267	130	16	664	162	233	61	4	527	69	0/99	0/884
Baye et al. (ii) [28]	35	140	140	210	420	12	25	14	49	53	0/89	0/48
Daneshmandi et al. [58]	63	15	3	141	21	94	26	4	214	34	0/2	0/862
Huang et al. [59]	1	19	80	21	179	4	43	75	51	193	0/46	0/209
Hwang et al. [60]	1	51	136	53	323	12	89	275	113	639	0/15	0/15
Chiang et al. [61]	13	110	329	136	768	7	34	65	48	164	0/38	0/226
Micheal et al. [62]	26	63	19	115	101	31	84	5	146	94	< 0.001	0/608
Ricciardolo et al. [63]	35	19	3	89	25	109	12	3	230	18	< 0.001	0/927
Smolnikova et al. [64]	36	28	0	100	28	39	11	0	89	11	0/38	0/89
Li et al. [65]	17	150	324	184	798	21	144	338	186	820	0/26	0/184
Wang et al. [66]	50	177	165	277	507	104	412	333	620	1078	0/17	0/365
Dahmani et al. [67]	13	19	12	45	43	6	11	2	23	15	0/35	0/605
Li et al. [68]	112	0	205	224	410	138	0	213	276	426	< 0.001	0/393
Narozna et al. [69]	117	55	5	289	65	133	53	3	319	59	0/37	0/843
Zhang et al. [68]	8	11	19	27	49	17	13	5	47	23	0/34	0/671
Hussein et al. [70]	42	5	1	89	7	8	13	4	29	21	0/73	0/58
Abood et al. [71]	66	17	17	149	51	7	90	3	104	96	< 0.001	0/52
Zhang et al. [72]	7	13	17	27	47	11	15	3	37	21	0/51	0/637

*P-HWE p*-value for Hardy–Weinberg equilibrium, *MAF* minor allele frequency of control group

### Meta-analysis of IL-4 SNP (C-589 T) and the risk of asthma

**Fig. 2:**
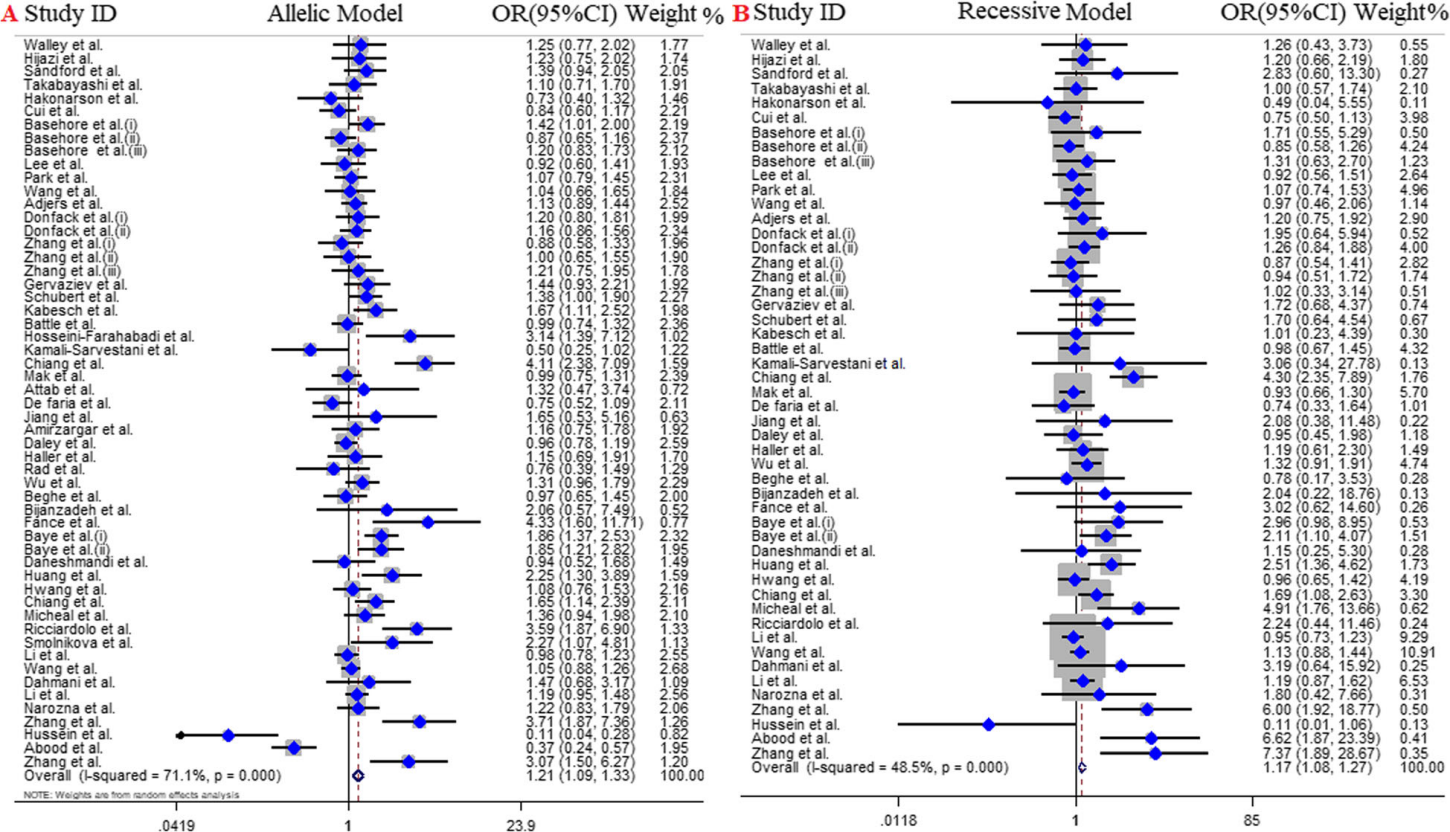
Pooled OR and 95% CI of individual studies and pooled data for the association between Il-4 C589T polymorphism and asthma risk in; **a** allelic model, **b** recessive Model

Overall, 55 studies with 9572 cases and 9881 controls included in quantitative analysis of the association between IL-4 gene -589C/T polymorphism and the risk of asthma. Of those, 11 articles were conducted in European countries [29, 31, 33, 38–41, 55, 63, 64, 69], 32 articles were in Asian countries [27, 30, 32, 34–37, 43–46, 49, 50, 53, 54, 56–62, 65, 66, 68, 70–73], 10 articles in American countries [16, 26, 28, 42, 48, 52] and one article in each Algeria [67] and Australia country [51]. The analysis of overall population revealed the significant positive association between *IL4* gene -589C/T polymorphism and the risk of asthma across all genetic models; including dominant model (OR = 1.22, 95% CI = 1.04–1.44, *P* = 0.01, REM), recessive model (OR = 1.17, 95% CI = 1.08–1.27, *P* < 0.001, FEM), allelic model (OR = 1.21, 95% CI = 1.09–1.33, *P* < 0.001, REM), and TT vs. CC model (OR = 1.34, 95% CI = 1.18–1.52, *P* < 0.001, FEM), except CT vs. TT model (OR = 1.13, 95% CI = 0.95–1.34, *P* = 0.17, REM) (Fig. 2). Additionally, along with subgroup analysis based on age we stratified the analysis by ethnicity in three conditions.

### Subgroup analysis by age

**Fig. 3:**
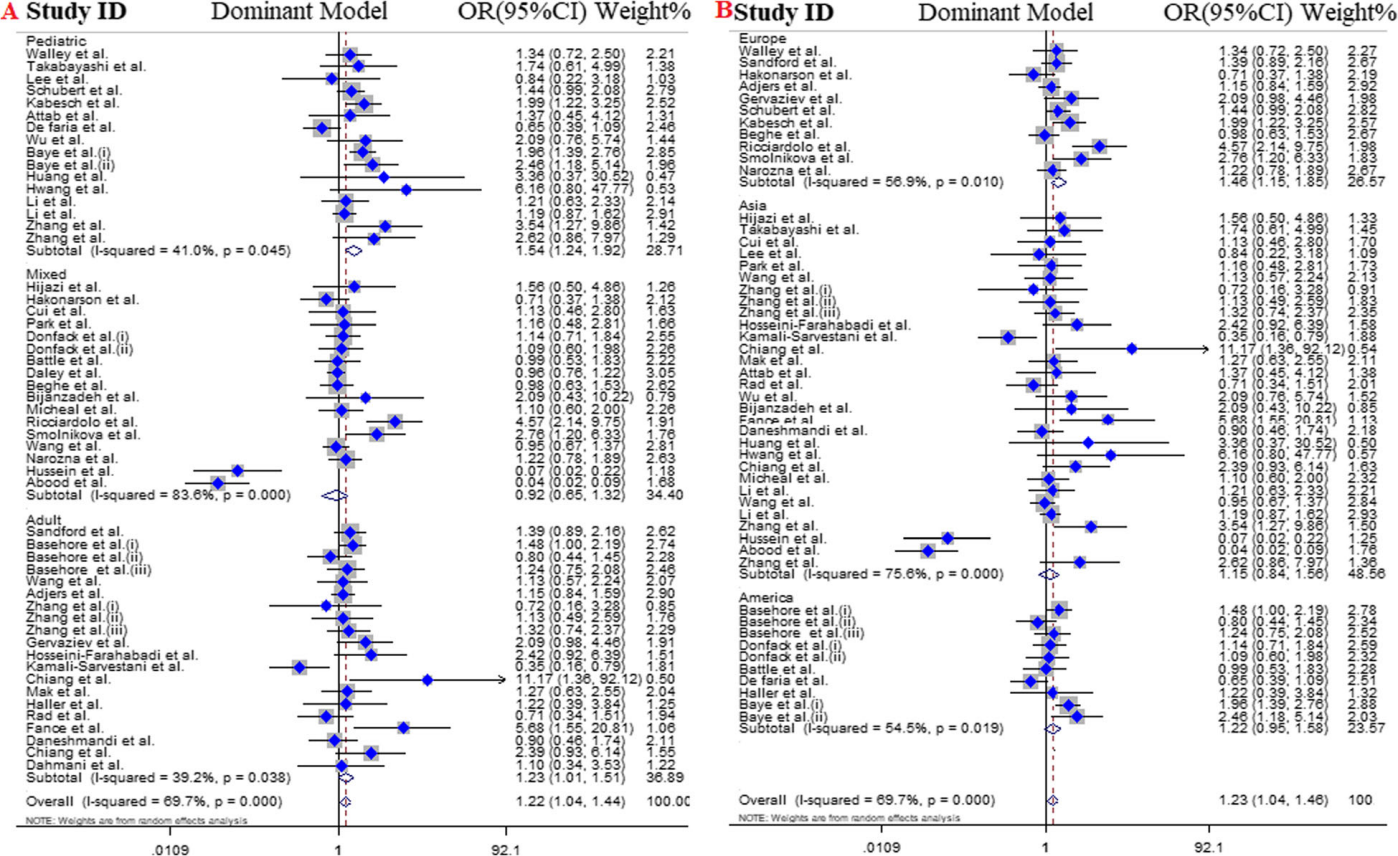
Pooled odds ratio and 95% confidence interval of individual studies and pooled data for the association between IL-4 C589T polymorphism and asthma risk in different subgroups for; **a** dominant model [age subgroup], **b** dominant model [continent]

We stratified eligible articles into three groups including: pediatrics (16 articles), adults (21 articles) and mixed (cover both range;18 articles). The results highlighted a predisposing role of *IL4* gene -589C/T polymorphism for the asthma risk in pediatrics and adults under all genotype models. However, no significant association was detected in mixed group **(**Table 3, Fig. 3).

**Table 3:** Main results of pooled ORs in meta-analysis of IL-4 gene polymorphisms in asthmatic patients

Subgroup		Sample size	Test of association		Test of heterogeneity		Test of publication bias (Begg’s test)		Test of publication bias (Egger’s test)	
	Genetic model	Case/Control	OR	95% CI (***p***-value)	I^**2**^ (%)	***P***	z	***P***	t	***P***
**Overall**	Dominant model	9579 / 9881	**1.22**	**1.04–1.44 (0.01)**	69.7	< 0.001	- 1.33	0.24	- 1.17	0.39
	Recessive model	9579 / 9881	**1.17**	**1.08–1.27 (< 0.001)**	48.5	< 0.001	−1.38	0.16	−0.60	0.55
	Allelic model	9579 / 9881	**1.21**	**1.09–1.33 (< 0.001)**	71.1	< 0.001	− 1.05	0.41	−1.82	0.07
	TT vs. CC	9579 / 9881	**1.34**	**1.18–1.52 (< 0.001)**	30.5	0.02	−1.25	0.24	−1.90	0.65
	CT vs. CC	9579 / 9881	1.13	0.95–1.34 (0.17)	68.7	< 0.001	−2.06	0.33	−1.73	0.09
**Age groups**										
**Pediatrics**	Dominant model	3061 / 3536	**1.54**	**1.24–1.92 (< 0.001)**	41	0.04	− 1.93	0.05	−1.63	0.23
	Recessive model	3061 / 3536	**1.20**	**1.05–1.37 (< 0.001)**	58.3	< 0.001	−0.36	0.71	−1.14	0.27
	Allelic model	3061 / 3536	**1.37**	**1.16–1.63 (< 0.001)**	68	< 0.001	−1.53	0.12	− 1.99	0.06
	TT vs. CC	3061 / 3536	**1.51**	**1.22–1.87 (< 0.001)**	51.6	0.01	−1.44	0.15	−1.47	0.24
	CT vs. CC	3061 / 3536	**1.49**	**1.23–1.81 (< 0.001)**	10.6	0.33	−1.92	0.05	−1.22	0.42
**Adults**	Dominant model	2933 / 2670	**1.23**	**1.01–1.51 (0.04)**	35.2	0.066	−2.10	0.03	−1.86	0.08
	Recessive model	2933 / 2670	**1.21**	**1.04–1.40 (0.01)**	46	0.01	−0.91	0.36	−0.71	0.48
	Allelic model	2933 / 2670	**1.24**	**1.05–1.47 (< 0.001)**	63.8	< 0.001	−0.97	0.33	−1.45	0.16
	TT vs. CC	2933 / 2670	**1.37**	**1.09–1.72 (< 0.001)**	5	0.39	−1.01	0.47	−1.77	0.19
	CT vs. CC	2933 / 2670	1.15	0.96–1.39 (0.13)	23	0.17	−2.13	0.03	−1.56	0.13
**Mixed**	Dominant model	3585 / 3675	0.92	0.65–1.32 (0.65)	83.6	< 0.001	−0.09	0.92	− 1.05	0.31
	Recessive model	3585 / 3675	1.12	0.97–1.28 (0.11)	45.4	0.02	−0.41	0.68	0.39	0.70
	Allelic model	3585 / 3675	1.03	0.85–1.24 (0.78)	76.3	< 0.001	−0.72	0.47	0.02	0.98
	TT vs. CC	3585 / 3675	1.14	0.91–1.42 (0.24)	20.8	0.21	−0.18	0.85	−0.28	0.87
	CT vs. CC	3585 / 3675	0.87	0.59–1.28 (0.48)	84.9	< 0.001	0	1	−1.11	0.28
**Ethnicity-1 (Continent)**										
**Asia**	Dominant model	5196 / 4936	1.15	0.84–1.56 (0.39)	75.6	< 0.001	−1.86	0.06	−1.44	0.20
	Recessive model	5196 / 4936	**1.16**	**1.06–1.28 (< 0.001)**	65	< 0.001	−1.62	0.10	−0.60	0.55
	Allelic model	5196 / 4936	**1.17**	**1–1.37 (0.04)**	76.7	< 0.001	−1.72	0.08	−1.04	0.30
	TT vs. CC	5196 / 4936	**1.34**	**1.13–1.58 (< 0.001)**	42.7	0.01	−1.48	0.13	−1.15	0.40
	CT vs. CC	5196 / 4936	1	0.70–1.42 (0.97)	75.1	< 0.001	−2	0.04	−1.42	0.20
**Europe**	Dominant model	1704 / 2347	**1.46**	**1.15–1.85 (< 0.001)**	56.9	0.01	0	1	−0.70	0.49
	Recessive model	1704 / 2347	1.35	0.98–1.86 (0.06)	0	0.94	− 1.58	0.11	−1.91	0.08
	Allelic model	1704 / 2347	**1.34**	**1.12–1.61 (< 0.001)**	51	0.02	−1.03	0.30	−1.50	0.16
	TT vs. CC	1704 / 2347	**1.53**	**1.10–2.14 (0.01)**	0	0.80	0.16	0.87	−0.87	0.40
	CT vs. CC	1704 / 2347	**1.44**	**1.13–1.83 (< 0.001)**	55.6	0.01	0.78	0.43	0.33	0.74
**America**	Dominant model	1991 / 1828	1.22	0.95–1.58 (0.11)	54.5	0.01	−1.33	0.27	−2.05	0.07
	Recessive model	1991 / 1828	1.15	0.96–1.39 (0.12)	24.3	0.22	−1.34	0.18	0.99	0.35
	Allelic model	1991 / 1828	1.19	0.99–1.44 (0.06)	64.8	< 0.001	− 0.98	0.32	−0.48	0.64
	TT vs. CC	1991 / 1828	1.27	0.98–1.64 (0.07)	43.7	0.06	− 1.52	0.12	−1.91	0.09
	CT vs. CC	1991 / 1828	1.18	0.94–1.48 (0.15)	39.3	0.09	−1.52	0.12	−1.94	0.08
**Ethnicity-2**										
**East-Asian**	Dominant model	4246 / 3908	**1.43**	**1.14–1.79 (< 0.001)**	26.3	0.14	−1.08	0.28	1.53	0.29
	Recessive model	4246 / 3908	**1.14**	**1.03–1.26 (< 0.001)**	66.6	< 0.001	−1.02	0.27	−1.51	0.36
	Allelic model	4246 / 3908	**1.29**	**1.10–1.52 (< 0.001)**	72	< 0.001	−1.79	0. 58	−3.10	0.06
	TT vs. CC	4246 / 3908	**1.33**	**1.11–1.59 (< 0.001)**	41.8	0.02	−1.27	0.29	−1.39	0.31
	CT vs. CC	4246 / 3908	**1.24**	**1.00–1.53 (0.04)**	0	0.74	−1.89	0.68	−1.71	0.10
**Non-East-Asian**	Dominant model	5333 / 5973	1.10	0.90–1.36 (0.35)	77.4	< 0.001	−0.80	0.42	−1.18	0.35
	Recessive model	5333 / 5973	**1.25**	**1.08–1.45 (< 0.001)**	21.9	0.14	0.59	0.55	0.73	0.47
	Allelic model	5333 / 5973	**1.15**	**1–1.32 (0.04)**	71.5	< 0.001	−1.05	0.48	−1.82	0.07
	TT vs. CC	5333 / 5973	**1.34**	**1.12–1.61 (< 0.001)**	24	0.11	−0.37	0.70	−1.04	0.30
	CT vs. CC	5333 / 5973	1.03	0.83–1.28 (0.78)	77.9	< 0.001	−1.16	0.24	−1.93	0.06
**Ethnicity 3**										
**Caucasian**	Dominant model	7360 / 7971	**1.30**	**1.12–1.51 (< 0.001)**	49.2	< 0.001	−1.04	0.48	−1.51	0.18
	Recessive model	7360 / 7971	**1.16**	**1.06–1.27 (< 0.001)**	49.7	< 0.001	−1.31	0.24	−2.77	0.09
	Allelic model	7360 / 7971	**1.25**	**1.12–1.39 (< 0.001)**	65	< 0.001	1.40	0.17	−1.12	0.38
	TT vs. CC	7360 / 7971	**1.34**	**1.16–1.56 (< 0.001)**	24.9	0.09	−1.52	0.16	−1.34	0.29
	CT vs. CC	7360 / 7971	**1.22**	**1.05–1.42 (< 0.001)**	39.6	< 0.001	−1.54	0.12	−1.80	0.08
**Arab**	Dominant model	316 / 284	0.36	0.07–1.88 (0.22)	91.5	< 0.001	0.68	0.49	−0.17	0.83
	Recessive model	316 / 284	1.53	0.27–1.48 (0.09)	87.4	< 0.001	0	1	−1.67	0.19
	Allelic model	316 / 284	0.63	0.67–3.68 (0.29)	85.4	< 0.001	0.49	0.62	−0.11	0.92
	TT vs. CC	316 / 284	0.93	0.43–1.99 (0.85)	66.6	0.02	0.68	0.49	1.25	0.33
	CT vs. CC	316 / 284	0.29	0.05–1.84 (0.19)	92.3	< 0.001	0	1	−0.71	0.55
**African-American**	Dominant model	1903 / 1626	**1.34**	**1.07–1.67 (0.01)**	35.3	0.13	−1.67	0.09	1.97	0.27
	Recessive model	1903 / 1626	1.18	0.98–1.43 (0.07)	24.7	0.22	0.63	0.53	1.11	0.30
	Allelic model	1903 / 1626	**1.25**	**1.04–1.50 (0.01)**	58.9	0.01	−1.46	0.14	−0.81	0.44
	TT vs. CC	1903 / 1626	**1.37**	**1.04–1.80 (0.02)**	36.2	0.12	−1.67	0.09	−1.44	0.40
	CT vs. CC	1903 / 1626	**1.30**	**1.06–1.58 (0.01)**	13.9	0.31	−1.67	0.09	−1.46	0.41

### Subgroup analysis by ethnicity 1 (continent)

In this subgroup we categorized studies by their continent: including Asia (32 articles), Europe (11 articles), America (10 articles), Africa (1 article), and Oceania (1 article). Since there was only one study for each one of the African and Australian population, these studies were excluded from the analysis. The results indicated that presence of *IL4* gene -589C/T SNP in Asian population increased susceptibility of asthma across all genotype models except dominant model (OR = 1.15, 95% CI = 0.84–1.56, *P* = 0. 39, REM) and CT vs. CC model (OR = 1, 95% CI = 0.70–1.42, *P* = 0. 97, REM). Moreover, in contrast with effect of *IL4* gene -589C/T SNP on the risk of asthma in American populations, a significant positive association was detected in European population thorough dominant model (OR = 1.46, 95% CI = 1.15–1.85, *P* < 0.001, REM), allelic model (OR = 1.34, 95% CI = 1.12–1.61, *P* < 0.001, REM), TT vs. CC model (OR = 1.53, 95% CI = 1.10–2.14, *P* = 0.01, FEM), and CT vs.CC model (OR = 1.44, 95% CI = 1.13–1.83, *P* < 0.001, REM) **(**Table 3, Fig. 3).

### Subgroup analysis by ethnicity 2 (east and non-east Asian)

**Fig. 4:**
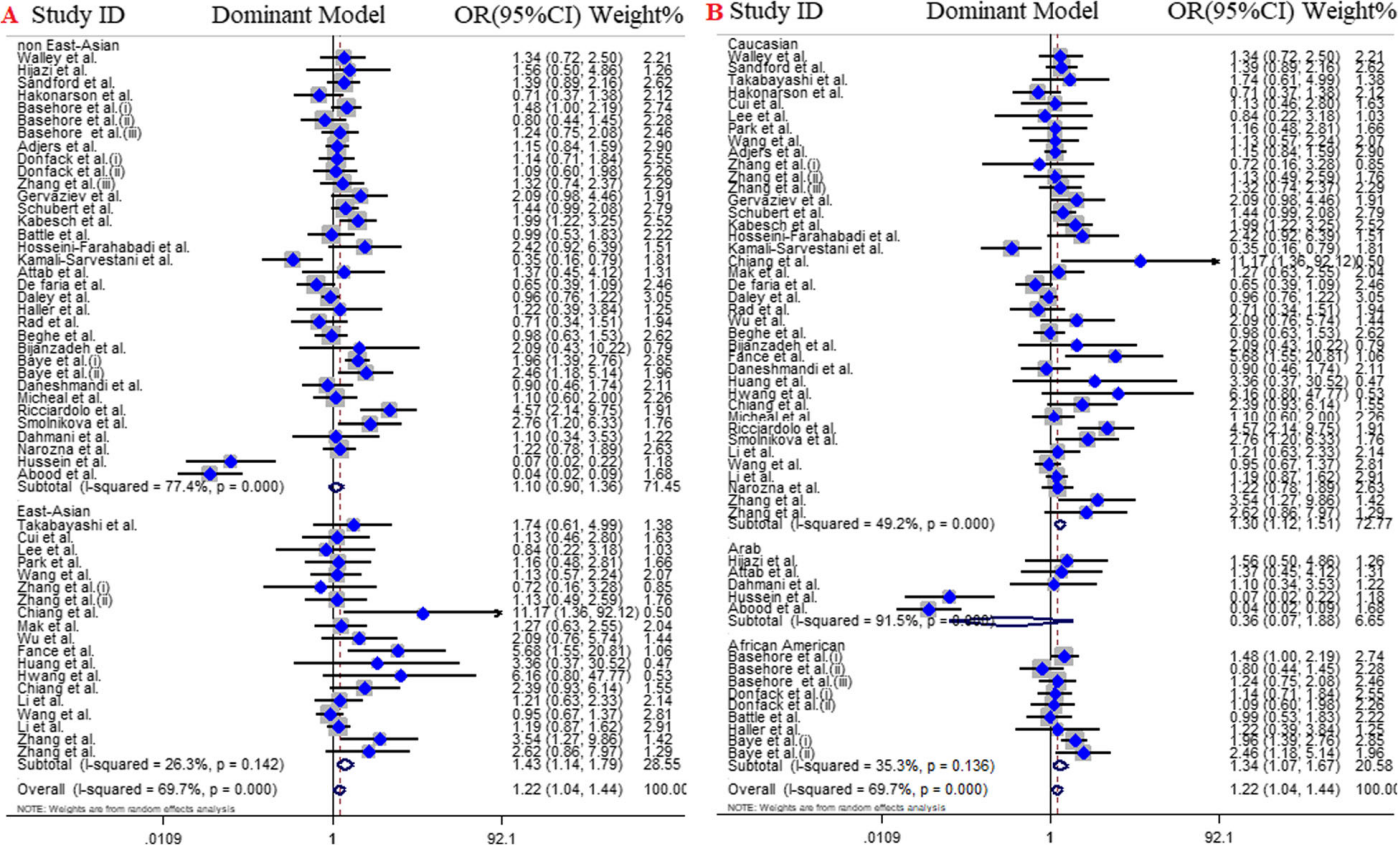
Pooled odds ratio and 95% confidence interval of individual studies and pooled data for the association between IL-4 C589T polymorphism and asthma risk in different subgroups for; **a** dominant model [East and non-East Asian], **b** dominant model [ethnicity]

The subgroup analysis according to East Asian (20 articles) and non-East Asian (35 articles) title revealed the significant association between *IL4* gene -589C/T polymorphism and the risk of asthma across in all genotype models of East Asians and three genotype models of non-East Asian including; recessive model (OR = 1.25, 95% CI = 1.08–1.45, *P* < 0.001, FEM), allelic model (OR = 1.15, 95% CI = 1–1.32, *P* = 0.04, REM), TT vs. CC model (OR = 1.34, 95% CI = 1.12–1.61, *P* < 0.001, FEM) (Table 3, Fig. 4).

### Subgroup analysis by ethnicity 3

Finally, subgroup analysis of eligible articles according ethnicity including Caucasians (41 articles), African-Americans (9 articles), and Arabs (5 articles) showed that there was no significant association between *IL4* gene -589C/T SNP and asthma risk in Arab population. Also, except recessive model (OR = 1.18, 95% CI = 0.98–1.43, *P* = 0.07, FEM) other genotype models in African-American population were significant including dominant model (OR = 1.34, 95% CI = 1.07–1.67, *P* = 0.01, FEM), allelic model (OR = 1.25, 95% CI = 1.04–1.50, *P* = 0.01, REM), TT vs. CC model (OR = 1.37, 95% CI = 1.04–1.80, *P* = 0.02, FEM), and CT vs. CC model (OR = 1.30, 95% CI = 1.06–1.58, *P* = 0.01, FEM). Conversely, all genotype models were significant in Caucasians and presence of *IL4* gene -589C/T SNP increase risk of asthma (Table 3, Fig. 4).

### Meta-regression analyses

**Fig. 5:**
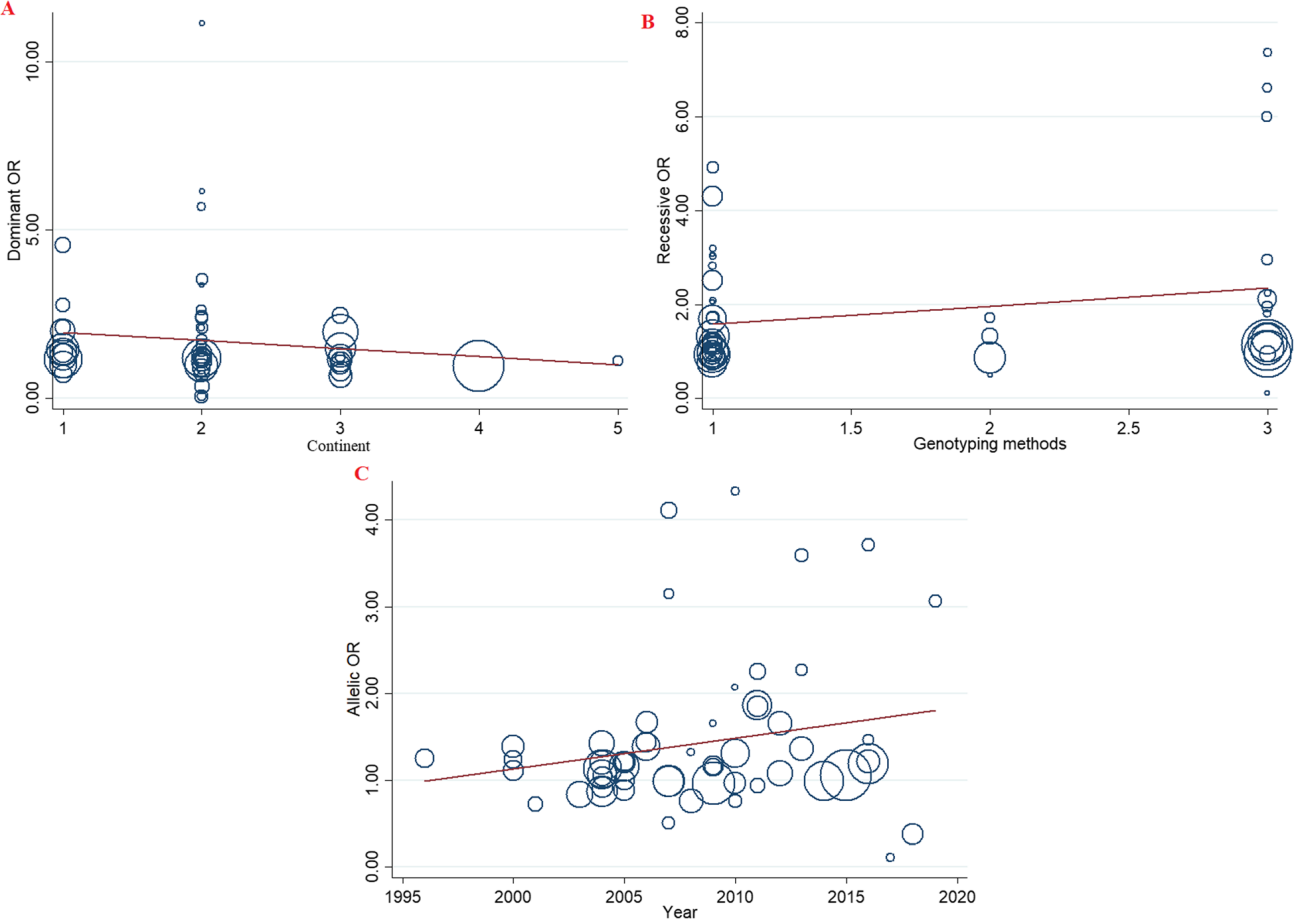
Meta-regression plots of the association between IL-4 C589T polymorphism and risk of asthma based on; **a** Continent (dominant), **b** Genotyping methods (recessive), **c** Publication year (Allelic)

Meta-regression analyses were performed to explore potential sources of heterogeneity among included studies (Table 4). The findings indicated that none of the expected heterogeneity parameter were the source of heterogeneity (Fig. 5).

**Table 4:** Meta-regression analyses of potential source of heterogeneity

Heterogeneity Factors		Coefficient	SE	T	***P***-value	95% CI	
						UL	LL
**Publication Year**	Dominant model	0.035	0.041	0.85	0.40	−0.048	1.119
	Recessive model	0.140	0.036	3.81	0.07	−0.066	0.213
	Allelic model	0.035	0.022	1.58	0.11	−0.009	0.080
	TT vs. CC	0.123	0.064	1.91	0.06	−0.006	0.254
	CT vs. CC	0.020	0.035	0.58	0.56	−0.050	0.090
**continent**	Dominant model	−0.238	0.265	−0.90	0.37	−0.772	0.294
	Recessive model	0.022	0.274	0.08	0.93	−0.530	0.574
	Allelic model	−0.116	0.146	−0.79	0.43	−0.410	0.177
	AA vs. CC	−0.096	0.435	−0.22	0.82	−0.973	0.780
	CA vs. CC	−0.265	0.209	−1.27	0.21	−0.685	0.154
**Genotyping methods**	Dominant model	−0.137	0.241	−0.57	0.57	−0.621	0.346
	Recessive model	0.382	0.232	1.65	0.10	−0.084	0.849
	Allelic model	0.039	0.130	0.30	0.76	−0.221	0.300
	TT vs. CC	0.056	0.388	0.14	0.88	−0.726	0.838
	CT vs. CC	−0.114	0.199	−0.57	0.57	−0.515	0.287

### Publication bias

**Fig. 6:**
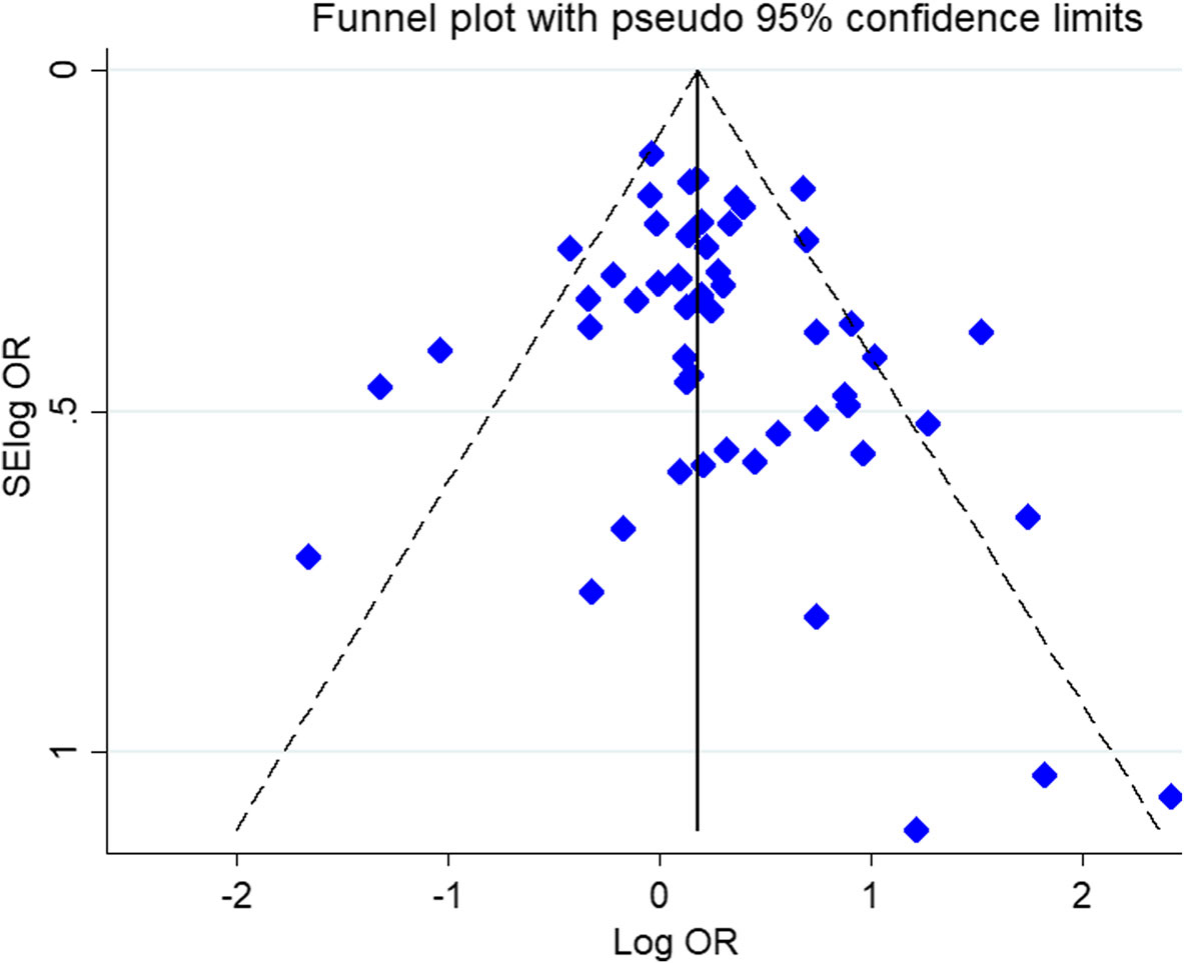
Begg’s funnel plot for publication bias test. Dominant model C598T. Each point represents a separate study for the indicated association

To check existence of publication, Egger’s linear regression and Begg’s funnel plot test were used. The shape of funnel plot did not disclose obvious asymmetry under all genotype model of the *IL4* gene -589C/T polymorphism (Fig. 6).

### Sensitivity analysis

**Fig. 7:**
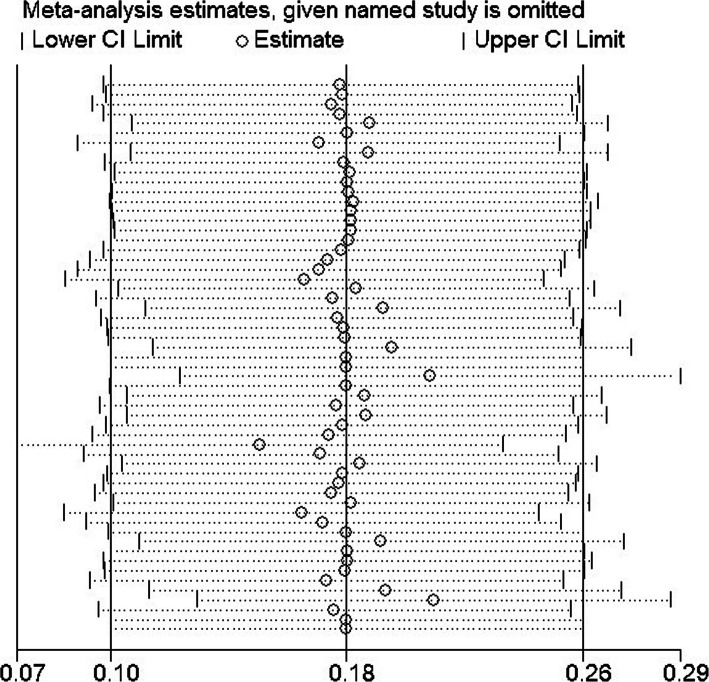
Sensitivity analysis in present meta-analysis investigates the single nucleotide polymorphisms of IL-4 C589T contribute to risk for asthma

The impact of individual study on pooled OR was evaluated by sequential omission of each studies. The result showed that no individual study significantly affected the pooled ORs under all genotype models of the *IL4* gene -589C/T polymorphism (Fig. 7).

## Discussion

To date, several individual case-control replication studies have attempted to divulge the association of *IL4* gene -589C/T polymorphism and risk of asthma. Due to some differences, however, these disperse investigation demonstrated incongruous reports. The differences in the race of study subjects, diversity in the diagnostic criteria of the patients, limited sample sizes may be the cause of such inconsistent results [74]. On the other hand, meta-analysis is a tool that has the potential to solve the problem of inconsistency by removing the confining issues of insufficient statistical power in the individual studies. Therefore, to resolve the mentioned confining factors about the *IL4* gene -589C/T polymorphism, the present most up-to-date meta-analysis was conducted to determine a bona fide estimation of the association between *IL4* gene -589C/T polymorphism and susceptibility to asthma. Our analysis indicated that this SNP was associated with increased risk of asthma in the overall population as well as during subgroup analysis by age groups and ethnicity/continent.

Asthma is a complicated pulmonary disease, characterized by airway hyperresponsiveness, airway inflammation, and airway remodeling [75, 76]. During asthma, there is a hyperactivity of Th2 responses, in which the cytokines of the type 2 immunity, such as IL-4, IL-5, and IL-13 promote the harmful inflammatory events in the airways. Studies have reported that local administration of IL-4 gene plasmids prior to antigen challenge could stimulate the airway hyperresponsiveness and accumulation of eosinophils in mice [77]. This phenotype of asthma is commonly referred to “eosinophilic” asthma. On the other side, “noneosinophilic” asthma is characterized by low frequency of eosinophils in the involved sites, but other inflammatory cells are dominant in the effector phase, such as neutrophils, mixed granulocyte inflammatory cells, or even little number of inflammatory cells, called paucigranulocytic inflammation. Th17 mediated IL-17 axis and lack of significant Th2/Th17 inflammation have been attributed to the noneosinophilic asthma [78]. Among the SNPs in the *IL4* gene, the -589C/T (rs2243250) polymorphism has been widely investigated in susceptibility to asthma. It has been shown that the T allele of this SNP leads to increased affinity of the binding of transcription factors in comparison to the C allele, leading to overexpression of IL4 mRNA [79, 80]. As a consequence, it is a biological justification that *IL4* gene −589C/T SNP impresses the IL-4 expression and, hence, could affect the asthma susceptibility.

Previously, three meta-analysis studies have attempted to disclose the association of *IL4* gene −589C/T SNP with the risk of asthma. Wang et al. in 2012 indicated that the T allele of *IL4* gene −589C/T SNP increased the risk of asthma (OR = 1.12). Basically, individuals carrying the T allele had a 24% increased risk of asthma in comparison to the CC homozygote model. Subgroup analysis revealed the association of this polymorphism in the Caucasians [81]. In addition, Nie et al. in 2013 included 40 studies involving 7345 cases and 7819 controls in their meta-analysis [18]. This meta-analysis indicated that TT vs. CC (OR = 1.40) and CT vs. CC (OR = 1.22) models were significantly associated with increased risk of asthma. In the subgroup analysis by ethnicity, significant associations were found among Asians and Caucasians, but not in the African-Americans. In addition, the subgroup analysis by atopic status revealed no significant association among atopic asthma patients and non-atopic asthma patients. On the other side, Zhang et al. [75] by evaluating pediatric asthma risk by evolving 17 case-control studies (15 publications) containing 3427 cases and 4247 controls revealed that IL4 -589C/T polymorphism was associated with increased risk of asthma in pediatrics. Furthermore, the subgroup analyses by ethnicity, indicated significant association in Caucasians and Asians.

Our analysis was performed on 55 case-control studies containing 9572 cases and 9881 controls. It was observed that *IL4* gene -589C/T polymorphism increased the risk of asthma across all genetic models, including dominant model (OR = 1.22), recessive model (OR = 1.17), allelic model (OR = 1.21), and TT vs. CC model (OR = 1.34), but not the CT vs. TT model. Furthermore, subgroup analysis by age indicated that *IL4* gene -589C/T polymorphism was significantly associated with asthma risk in both pediatrics and adults. The subgroup analysis by ethnicity revealed significant association in Asian, American, and Europeans. Finally, subgroup analysis by East Asian and non-East Asian populations indicated significant associations.

This meta-analysis bears some limitations and caveats. First, the analysis was according to crude estimation of *IL4* gene -589C/T polymorphism association with asthma susceptibility, regardless of the effect of confounding factors, like age, sex, environmental factors, and contribution of other genes in LD with *IL4* gene. Second, we did not analyze other genes that could be contributing in understanding of cytokine involvement in the susceptibility to asthma.

## Conclusion

All in all, here we carried out the most up-to-date analysis of the *IL4* gene 589C/T polymorphism and asthma risk prior to September 2020. Our meta-analysis further confirmed some results of the previously performed meta-analysis, while rejected some of them. In a whole, *IL4* gene -589C/T polymorphism increased the risk of asthma across all genetic models. Moreover, the subgroup analysis by age indicated that *IL4* gene -589C/T polymorphism was significantly associated with asthma risk in both pediatrics and adults. Also, the subgroup analysis by ethnicity revealed significant association in Asian, American, and Europeans. Ultimately, subgroup analysis by East Asian and non-East Asian populations indicated significant associations.

## Data Availability

All data that support the conclusions of this manuscript are included within the article.

## Abbreviations

IL: Interleukin
Th: T helper
CI: Confidence interval
OR: Odds ratio
SNP: Single-nucleotide polymorphism
PRISMA: Preferred Reporting Items for Systematic reviews and Meta-Analyses
NOS: Newcastle–Ottawa scale
HWE: Hardy–Weinberg equilibrium
